# Personal Network Analysis in the Study of Social Support: The Case of Chronic Pain

**DOI:** 10.3390/ijerph15122695

**Published:** 2018-11-29

**Authors:** Rosario Fernández-Peña, José Luis Molina, Oliver Valero

**Affiliations:** 1 Department of Nursing, SALBIS Research Group, Nursing Research Group IDIVAL, University of Cantabria, 39008 Santander, Spain; 2 Department of Social and Cultural Anthropology, GRAFO, Universitat Autònoma de Barcelona, 08193 Barcelona, Spain; joseluis.molina@uab.cat; 3 Servei d’Estadística Aplicada, Universitat Autònoma de Barcelona, 08193 Barcelona, Spain; oliver.valero@uab.cat

**Keywords:** social support, social networks, chronic illness, chronic pain, personal networks

## Abstract

In the context of chronic illness, the individual’s social and relational environment plays a critical role as it can provide the informal support and care over time, beyond healthcare and social welfare institutions. Social Network Analysis represents an appropriate theoretical and methodological approach to study and understand social support since it provides measures of personal network structure, composition and functional content. The aim of this mixed method study is to present the usefulness of Personal Network Analysis to explore social support in the context of chronic pain. Personal and support network data of 30 people with chronic pain (20 alters for each ego, 600 relationships in total) were collected, obtaining measures of personal network structure and composition as well as information about social support characteristics. Also, semi-structured interviews with participants were conducted to identify the context of their experience of pain, their limitations as regards leading an autonomous life, their social support needs and other aspects concerning the effect of pain on their social and relational lives. This approach shows the importance of non-kin social support providers and the significant role of non-providers in the personal networks of people suffering chronic pain.

## 1. Introduction

Chronic pain is a major public health problem due to both its prevalence and its significant effects on patients, their families and the social and professional environment, leading to a deterioration in the quality of life [1]. Prevalence data for chronic pain obtained from population-based surveys vary considerably. Global estimates range from 2% to more than 55% and from 14.6% to 64% in the United States, 19% of adults are affected in Europe and 16.6% of the adult population in Spain [2,3,4], with a higher prevalence among women and older adults. Pain has been defined by the International Association for the Study of Pain (IASP) as an unpleasant sensory and emotional experience associated with actual or potential tissue damage, or described in terms of such damage [5], and is considered one of the most complex human experiences. Also, it is subjective in nature, a leading cause of stress and the most frequent reason for medical consultation. The biopsychosocial model, considered essential in the occurrence of pain, provides a framework to understand how biological, psychological and social processes interact and affect pain significantly [1]. This model recognises the role of family, work and social networks in its social dimension. Thus, it is necessary to investigate the individual’s personal relationships within the most immediate social environment and the resources derived from these social ties.

One of the resources derived from social ties is social support, a concept that has attracted considerable attention and has been explored from a variety of perspectives, with particular emphasis on different dimensions, components and mechanisms of action. A widely used definition of this concept was proposed by Cobb in 1976: “Social support is […] information leading the subject to believe that he is cared for and loved, esteemed, and a member of a network of mutual obligation” [6] (p. 300). Its conceptual development has built a variety of definitions whose suitability and usefulness are to a large extent mediated by the context in which social support operates and is studied [7]. In addition to this, empirical work on social support began in the 1970s following studies by the epidemiologists John Cassel and Sidney Cobb [6,8], who established a relationship between social support and the risk of illness for the first time. They found that social support exerted a protective effect against stress and also influenced susceptibility to illness. These initial mechanisms of influence formed the basis for including social support in larger unifying models.

The impact of social relationships that provide support on health has been well established in the literature. Researchers have used many general and interchangeable terms, including social networks, social ties, social integration and other constructs such as social support [9]. Nevertheless, it is necessary to distinguish between social support and social networks, understanding the latter as the structural framework in which social support is available or not to an individual [10]. In their review of social support and health, House et al. [11] proposed two elements of the relational structure that are involved in social support: social integration, which refers to the existence or amount of social relationships and social network structure, which refers to the structural properties characterising a group of relationships. Berkman et al. [9] developed a conceptual model using a cascade of factors to explain how social networks influence health. In this model, the conditions of the social structure at macro-level determined by socio-economic, cultural or political factors influence the extent, shape and nature of social networks at meso-level. In turn, the network structure characteristics (e.g., density or size) and the social ties they contain (e.g., frequency of contact, duration or multiplexity) create the framework for relational and psychosocial mechanisms such as social support.

As part of the development of social support as a concept, it needs to be highlighted the operationalisation of various dimensions such as types of support [12,13,14], the difference between received and perceived social support [15,16] and the importance of the context in which it operates and the meaning attributed to it [17]. Types of social support have constituted one of the most widely studied dimensions due to their relationship with different types of stressor. Thus, the main types of social support are: emotional support (behaviour that fosters the feeling of comfort and leads a person to believe that he or she is admired, respected and loved, and that others are available to provide care and safety), informational support (knowledge, advice or information, which helps an individual to understand his or her world and adapt to changes that occur in it), and instrumental support (material or tangible support in terms of goods and services that help solve practical problems) [13]. Other related concepts are: functional social support, (the varied kinds of resources that flow through the network’s social ties), social networks (a unit of social structure composed of the individual’s social ties and the ties among them) and structural social support (the number and pattern of direct and indirect social ties that surround the individual) [18].

A substantial increase in health studies that use Social Network Analysis approaches has witnessed in the last decade [19,20,21,22,23]. They analyse structural and relational aspects of health, being relevant to stakeholders in order to improve well-being, health behaviours and social support [24,25,26]. In this sense, the application of social networks approach in the study of social environments where people live and work is fundamental to understand how interactions, social connectedness, and functions and types of social networks might be critical for health outcomes at different times and ages [27,28]. Some authors have established types of networks based on the characteristics of network composition or types of social support or interaction such as frequency, and these have been related to aspects such as the capacity of interpersonal environments to mobilise and share resources [29], health-related behaviours [30], perceived availability of social support [31] and healthcare usage [32]. The main idea of this approach is that individuals are surrounded by a network of relationships that influence health-related behaviours [33] and help-seeking resources. Therefore, our health is interconnected with our relationships [34]. In the field of health, Social Network Analysis constitutes a suitable methodology to answer research questions that involve relational data and can be characterised as follows: (i) it is a structural approach focused on the patterns of ties between actors; (ii) it is based on empirical data; (iii) it makes frequent use of mathematical and computational models; and (iv) it is highly visual since network data can be represented by means of graphs [19]. Traditionally, two approaches have been distinguished in Social Network Analysis. On the one hand, the sociocentric perspective focuses on the pattern of ties within a socially defined group. On the other hand, the ego-centred perspective focuses on the social relations of individuals (ego). The Analysis of Personal Networks, which is the focus of the present study, forms a subset of the broader concept of ego-centred networks and targets on the relationships surrounding individuals in all the social environments to which they belong (e.g., family, co-workers and neighbours) [35,36].

Many instruments have been developed for the measurement and assessment of social support since it was first defined over four decades ago. These instruments explore the presence of varied types of social support and social network characteristics (e.g., size and network member characteristics), focusing mainly on the significant ties that provide support for the individual [10,37,38]. Notwithstanding, these instruments have largely been based on the analysis of the dimensions of social support, viewing them as attributes of the people surrounding the individual, without considering the structural and relational aspects of the individual’s personal environment. From this perspective, Personal Network Analysis constitutes an excellent approach to study social support since it can be used to measure the structure and composition of personal networks and the functional content of social support. This approach is based on collecting data from an informant (ego) who provides information on his or her relationships, naming the people in his or her social environment (alters), describing their characteristics and attributes and indicating the relationships between them. Thus, the main aim of this study is to present the usefulness of Personal Network Analysis for identifying individual’s social and relational environment regarding the complex process of providing and receiving social support in the context of chronic pain.

## 2. Materials and Methods

### 2.1. Design

Cross-sectional design with mixed methodology combining Personal Network Analysis and semi-structured interviews.

### 2.2. Sample Description

Participants comprised 30 people (15 men and 15 women) diagnosed with chronic pain and receiving care at Marqués de Valdecilla University Hospital Pain Unit (Santander, Spain). Fieldwork and analyses were conducted between July 2014 and July 2015. Participants were selected through convenience sampling during visits or outpatient treatment for pain. Data were collected at the hospital or informants’ homes following their health status and personal preferences. Results on the personal networks of the 30 cases studied and 600 personal relationships (20 relationships per ego) were obtained.

### 2.3. Variables

Personal network:

(a) Personal network structure: unlike sociocentric or complete networks, in which each member is considered to contribute equally to shaping the network structure, personal networks examine the influence on the informant (ego) of each member of a network, based on the principle that not all network members contribute equally to the phenomenon under study [35], be that behaviour, personality or social support. Personal network data were obtained regarding:
  Density: the percentage of ties that exist in a network out of all possible ties.
  Degree centrality (two measures: mean of a personal network and at node level): number of vertices adjacent to a given vertex in an asymmetric graph or the number of relations of a given person. It is a measure of network activity. An alter is highly degree central to the extent that he or she is directly connected to many other alters.
  Betweenness centrality (two measures: mean of a personal network and at node level): number of times a vertex occurs on a geodesic (e.g., the shortest path) connecting every other pairs in a graph. A single alter is highly between central to the extent that they lie on many geodesics (shortest paths) between alters. In this sense, they act as a bridge between alters and thus, potentially, control information, for example.
  Components: set of alters who are connected to one another directly or indirectly.
  Isolates: actors who are not connected to any other actors.
(b) Personal network composition (alter characteristics): age, sex, type of tie with ego, place of residence and proximity.
(c) Social support (function): type, satisfaction, reciprocity, variation over time, frequency and channel of transmission. In this specific context of chronic pain, support was considered generally: emotional support, such as the interpersonal transactions that make a person feel understood and accompanied in his or her pain; instrumental support, such as the help of other people in activities that the individual either cannot perform or for which others are required as a result of pain (e.g., help in mobility, hygiene, clothing or personal care); and informational support, such as advice or information about chronic pain that helps the subject deal with the pain (e.g., formal support resources or self-help groups and associations).

### 2.4. Data Collection Instruments

Personal network: EgoNet open source software (https://sourceforge.net/projects/egonet/ v.2014) was used to collect and analyse each ego’s personal network data. Also, UCInet software [39] was used to obtain degree and betweenness centrality for each of the 600 alters studied. Personal network data were collected in four stages based on an ad hoc questionnaire designed following the study aim:

First stage: ego data collection (e.g., age, sex, educational level, date of onset of pain).

Second stage: informants were asked to nominate the people who formed part of their social and relational life using a question to generates names (known as “name generator”)*:* Please name 20 people in your social environment that you know by name and vice versa, with whom you have had at least some contact in the past year and that it would be possible to contact if necessary. Try to include all the environments of your social life.

Third stage: informants were asked about the alter’s characteristics (personal network composition) and variables related to functional aspects of social support (social support function) for each of the nominated contacts. Eleven questions about each of the 20 alters nominated were asked to the informants.

Fourth stage: informants were asked about the relationship between possible pairs of actors from among the contacts nominated.

In comparison with other instruments for assessing social support, a Personal Network Analysis, has some advantages:

(a) Uses a name generator able to elicit a large number of alters that distinguishes between the global personal network and the specific social support network. It allows to identify non-providers and their characteristics in terms of attributes and their position in the network structure.
(b) Facilitates an analysis of different social support dimensions:

The structural dimension of the personal network: variables that reflect network cohesion or fragmentation, such as density, centrality, or the number of components or isolates [28].

The functional dimension of social support based on the informant’s subjective assessment of each of the alters’ social support attributes, whether providers of support or not (e.g., type of support, satisfaction, frequency).

The contextual dimension, through visual representation of the data in a graph, which has a high explanatory power. Visualisation of the personal network is a key tool that condenses large amounts of information into a single visual representation. In addition to visualisation, qualitative techniques such as interviewing informants about network relationships, constitute an extraordinary resource for working with informants to explore the meaning of their personal and support reality [40,41], which is especially useful in the context of illness.

(c) Facilitates the simultaneous study of micro phenomena (interactions) and meso phenomena (local structures surrounding informants, including institutions) [42], which in the specific case of social support can reveal the existence of formal social support structures such as support groups, voluntary organisations or social resources in the community, besides the support provided mainly by family, friends, neighbours and other personal contacts.
(d) Determines personal network structure and composition and the characteristics of the social support contained in relationships, enabling simultaneous study of other health outcomes (e.g., quality of life, stress).

Semi-structured interviews: they were used in order to identify the context of their experience of pain, their limitations, their social support needs and other aspects concerning social support in their personal relationships as well as the effect of pain on their social and relational lives.

### 2.5. Data Analysis

Interviews were audio-recorded using Windows Media Encoder v.9 (https://www.microsoft.com/es-es/download/details.aspx?id=1659) and transcribed verbatim. The information was analysed through thematic categorisation and encoding. Based on the initial interviews, new sub-themes were identified that were then included in the analysis by assigning new sub-codes.

A descriptive analysis was conducted for informants’ sociodemographic and pain descriptors, variables related to personal network structure and composition and social support characteristics. Counts and percentages were estimated for qualitative variables and mean, median and minimum and maximum deviation were calculated for quantitative variables. All statistical analyses were performed using Statistical Analysis Software (SAS) software v9.3 (SAS Institute Inc., Cary, NC, USA).

### 2.6. Ethical Considerations

This study was approved by the Clinical Research Ethics Committee of Cantabria (internal code 2014.32). Participants received verbal and written information about the aim and procedure of the study and gave their signed informed consent once they had decided to participate on a voluntary basis. Throughout the study, national and international guidelines (Code of Ethics and Declaration of Helsinki) were followed and data confidentiality legislation (Spanish organic law 15/1999 of 13 December on the protection of personal data) was observed.

## 3. Results

### 3.1. Sociodemographic and Pain Descriptors

The mean age of participants was 54.57 years (SD: 11.64, range 30–73 years). Their marital status was: married or with partner (N = 27); divorced (N = 1); and widowed (N = 2). Their educational level was: primary education (N = 16), vocational education (N = 8), secondary education (N = 4) and higher education (N = 2). At the time of the interview, their employment status was: active (N = 9), on sick leave due to pain (N = 6), retired (N = 10) and housework (N = 5).

Mean pain intensity at the time of the interview was assessed using a numerical scale from 0–10, was 5.87 for men (SD 2.03, range 2–8) and 6.33 (SD 2.13, range 2–9) for women. Given the chronic nature of pain and its variation over time (in general, periods of stability alternating with flare-ups), informants were asked to indicate the maximum level of pain they had experienced since its onset. The results show that of the 30 participants, 22 had experienced the maximum pain level of 10 at some point in time. The mean length of time since onset of chronic pain was 12.2 years (SD 9.18, range 1–35 years) in men and 16.6 years (SD 12.39, range 1–39) in women. The most frequent location of pain reported by the informants was the back (neck, back and lower back) and was related to disc problems and arthrosis.

### 3.2. Experience of Pain

The informants’ accounts of their experience of pain showed that it affected several areas of life, including the social and relational dimension. Most of the informants’ accounts indicated reduced participation in social leisure activities, weakened social ties and a tendency towards social isolation both of themselves and of those closest to them:

The pain has stopped me from living, from having a social life, from working, from being with my family, from being with my friends, because the pain stops me from getting out of the house. I don’t have a life, I go from bed to the sofa and from the sofa to bed. And being at home so much drives you crazy. Because days go by, and they’re days of your life that serve no purpose. Man, 47 years old.

I never go out, not to eat with friends, not to go for a walk, not … not even to go for a walk with my child … this last year I haven’t been out at all. Woman, 31 years old.

[…] You can’t plan or do anything, it’s … it tortures you, you get depressed, you … well I was begging them to amputate my leg as soon as I knew nothing could be done […], it’s devastating to be in so much pain, not knowing what to do, not knowing how to act … it destroys you: as a person, as a woman, as a worker, as a mother, as a wife … it completely annihilates you. Woman, 46 years old.

When I have one of my very bad days I, I … how can I say it? I don’t want to be with them. I don’t, because I feel so unwell, nothing feels good, everything looks black, and I need to be alone […]. I’m very moody. It makes me feel so powerless, I can’t do anything and nobody can do anything for me. Woman, 66 years old.

I used to go out, go walking with my friends, I looked after my daughter [cries] … it’s looking after my daughter that’s the worst thing because … I can’t give her a bath because I’d have to bend over and I can’t, fetch her from school … that’s the worst thing for me. Woman, 34 years old.

I used to have a very wide circle but not now, not for some years now, there’s no circle. Only the very closest ones are left, the people at home. Things happen to you, as they did to me, and you lock yourself away with your pain and misery, because you can’t get around like you did before […]. It’s made me distance myself in the sense of not wanting to go out. Man, 60 years old.

Other accounts concerned the negative effects of pain on the family environment due to behavioural changes caused by pain, the consequences for family dynamics and the impact of pain on other members of the family:

It affects everything around me, everything, absolutely everything. In general, friends … I don’t do much because I’m not up to it, because actually I have 8 brothers and sisters [cries], and I realise that I can’t spend an hour sitting down, I can’t lead a life like … I’d love to spend time with them. Woman, 52 years old.

The people around me, mainly. I didn’t realise until I saw that they were suffering as much as I was when they saw the state I was in (…). Yes, those around me, I understand because somehow I get … I snap and don’t realise until later … I not as patient as I ought to be, sometimes I get aggressive and say things I wouldn’t normally say given my education and my way of being … (…) and then I feel like I’m someone else, I’m not myself, and that really upsets me. Man, 65 years old.

[…] sometimes very bad, sometimes I realise that I’ve been taking it out on the first person to cross my path. And feeling ill, sometimes anything they said to me would set me off. Because I wasn’t well … you know? Yes, of course it changed me, of course it did. Woman, 66 years old.

[…] Sometimes you have to say no to certain things, because I can’t, or I can but I don’t want to because I’ll suffer for it later, so of course you limit yourself a bit … you have less social life at times, and not just you but those closest to you as well because they’re not always going to say “Well you stay at home and we’ll go”, but … Man, 46 years old.

The effects of pain regarding limiting activities of daily living were also mentioned in the interviews, indicating the need for support or help from the personal environment:

For the two months I was in bed, I was completely helpless because they had to pick me up, carry me to the shower, wash me and then dress me and put me back to bed. Once they gave me injections I could do it for myself, but they had to sit me on a seat in the shower in order for me to be able to wash myself. I couldn’t stand. Man, 47 years old.

Housework: my husband has to help me make the bed or sometimes he has to do it himself because I can’t. Sometimes it’s the same for getting dressed, I have to tell him: help me tie this because I can’t, or get me that because I can’t, or do this because my hands aren’t strong enough. Woman, 66 years old.

When I got ill they were the ones who really helped me. They helped me a lot because sometimes I couldn’t get out of bed, I couldn’t cook […], I couldn’t even dress myself, I couldn’t do anything because the pain was so strong, I wouldn’t wish this on anyone, honestly, it was so horrible that … Man, 30 years old.

The accounts of pain that were collected clearly evidenced its multidimensional nature, rendering it a total experience. Similarly, its chronic nature, in which more stable periods alternated over time with periodic flare-ups, highlighted the importance of the personal environment in providing informal support and care.

### 3.3. Descriptive Analysis

#### 3.3.1. Personal Network Composition Variables

The personal contacts nominated by the informants consisted of 310 (51.7%) women and 290 (48.3%) men with a mean age of 48.3 years (SD:18.6, range 1–92). By sex, women alters presented a mean age of 49.5 years old and men, 47.11. Sex was fairly evenly distributed, with women alters accounting for 51.67% (N = 310) and men, 48.33% (N = 290). According to the sex of the egos, women had more women in their networks (64%) than men (40%). The alters’ ties with the ego presented the following distribution: spouse (N = 25, 4.2%), parent (N = 16, 2.7%), sibling (N = 65, 10.8%), child (N = 61, 10.2%), other relative (N = 180, 30%), friend (N = 177, 29.5%), neighbour (N = 38, 6.3%), co-worker (N = 29, 4.8%) and relationship established because of the pain (N = 9, 1.5%). Tie strength defined as a function of proximity presented the following distribution: very close (N = 137, 22.8%), fairly close (N = 150, 25%), close (N = 158, 26.3%), not very close (N = 109, 18.2%) and not close at all (N = 46, 7.7%). With regard to the alters’ place of residence, most contacts lived in the same town as the ego (N = 264, 44%) or in the same province (N = 236, 39.3%), although some lived in another province (N = 80, 13.3%) or in another country (N = 20, 3.4%).

#### 3.3.2. Personal Network Structure Variables

The results for personal network structure (see Table 1) did not show significant differences according to sex of the ego, although a trend was observed among women to have more cohesive, less fragmented personal networks with greater betweenness than men.

These differences in personal network structure and composition together with other contextual variables might explain, at least in part, the social support content presented below.

#### 3.3.3. Functional Variables of Social Support

In the analysis of 600 relationships, 401 support providers (66.8%) and 199 non-providers (33.2%) were identified. The analysis of social support characteristics (see Table 2) showed that the quality of social support presented a variable distribution, with different percentages of relationships classified as very satisfactory (12.3%), quite satisfactory (22%), satisfactory (26%), quite unsatisfactory (6.5%) and very unsatisfactory (33.2%), where the latter included non-providers. 

Due to the chronic nature of the pain, variation in social support over time is particularly important. Most of the relationships analysed did not show variation in the social support they provided. Notably, the percentage of relationships where support had increased or decreased was similar. In addition, social support had been reciprocal in most of these relationships (the ego had offered some kind of support to the alter). In relationships providing social support (N = 401), this was primarily emotional or emotional and instrumental and was generally provided weekly or daily, in person or in person combined with telephone calls as the channel of transmission.

#### 3.3.4. Relationships not Providing Social Support

The mean number of support providers in the networks studied was around 13 people, with a variable degree of satisfaction within the support that these relationships contained and transmitted, and 7 non-providers, with a similar percentage in women’s (31%) and men’s (36%) networks.

In terms of composition, relationships not providing support were characterised by:

- Sex: more men (N = 115, 57.79%) than women (N = 84, 42.21%) were non-providers.
- Age: non-providers presented a mean age of 46.46 years old, but encompassed both ends of the life cycle (range 1–92 years).
- Type of tie: non-providers included friends (N = 64, 32.16%), family (N = 48, 24.12%), other relatives (N = 35, 17.59%) and close family (N = 28, 14.07%), including 3 spouses, 15 siblings and 10 children. Parents formed the only social tie not represented among non-providers. To a lesser extent, neighbours (N = 13, 6.53%) and co-workers (N = 11, 5.53%) appeared among the non-providers.
- Reciprocity: the non-provider group included a higher percentage of relationships in which the ego had provided support (N = 118, 59.3%) than those in which the ego had not done so (N = 81, 40.7%).
- Variation in support over time: the absence of support was maintained over time in almost all non-provider relationships (N = 183, 91.96%), and support decreased in a small percentage of cases (N = 16, 8.04%).
- Tie strength: non-providers of support presented a weak tie with the ego (N = 101, 50.75%), although notably, the ego presented a strong tie with a similar percentage of these contacts (N = 98, 49.25%).
- Place of residence of the alter: most non-providers of support resided in the same town as the ego (N = 86, 43.22%) or in the same province (N = 80, 40.20%). A smaller percentage resided in another province (N = 25, 12.56%) or in another country (N = 8, 4.02%).

Adding to these quantitative results, the qualitative information obtained indicated that the elderly and very young did not provide support due to characteristics associated with age. Illness of contacts was another reason for non-provision of support. In other cases, informants cited lack of understanding or empathy regarding their pain among the reasons given in their accounts for the non-provision of support, and this arose in personal relationships with close family members such as spouses, children or siblings.

I’m a househusband and nurse because my wife is bedridden, she’s ill. […]. No, the children are only concerned with work, enjoying themselves, asking for something, but everything else? At least with me. […]. Man, 66 years old.

Let’s see, he does help me a lot [her husband], because he’s there but in my heart I don’t think he understands either… I mean, he understands that I’m in pain but at the same time he’s also very like…: well, you’ll have to cope, there’s no alternative. […] I’d like more [referring to support], I’d like much more but I can’t force people … Women, 34 years old.

[…] I’d have expected more support from her [her daughter]. She doesn’t understood me enough, either (…) I expected more support from her and more help. I think it might be because she doesn’t really believe me, because she’s also going through a bad patch […]. I’d like to think that she’s upset for me, I mean, that she doesn’t know how to help me but she does know how I am from the doctors, who’ve told her “your mother is very ill and so on”, but she doesn’t give me that impression. Maybe it’s because I don’t say anything and I need people to say it to me. Woman, 56 years old.

Yes, there’s a relationship but it’s not support … it’s different, no. We’re family but… there isn’t a relationship of, of, of … you often have more contact, more support, with a neighbour than with a family member. […] We’re sisters and we see each other almost every day but it’s not to say: you’re ill and I’m going to support you … no, no, no. I’ve had several operations and she’s never been to visit and so on, no, no. Woman, 66 years old.

With regard to structure, the means for degree and betweenness centrality of non-providers and for all alters are given in Table 3, showing that the former present lower means than the latter.

These results identify non-providers of support as alters with fewer ties with other members of the network (degree centrality is lower than the total mean) and mediating or forming a bridge between other alters to a lesser extent than the alters as a whole (betweenness centrality is lower than the total mean). Thus, from a structural perspective, non-providers of support are located on the periphery and distant from the core of a network, although it is important to consider other contextual factors which in some cases might explain the non-provision of support (for example, an alter very close to the ego and with a high degree and betweenness whose age or health status could explain the non-provision of support).

### 3.4. Visualisation of Personal Support Networks

Graphs are visual representations of networks, displaying actors as nodes and the relational ties connecting actors as lines. By way of example, this section presents graphs of personal support networks, showing the diversity of relational and functional contexts of social support in the cases studied.

**Figure 1:**
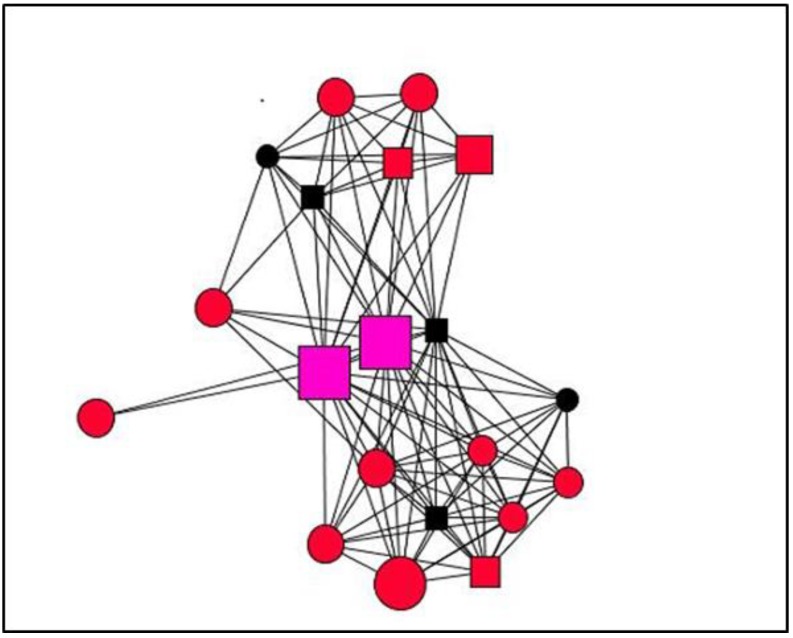
Graph Case 2. Women, 31 years old, 8 years with chronic pain.

**Figure 2:**
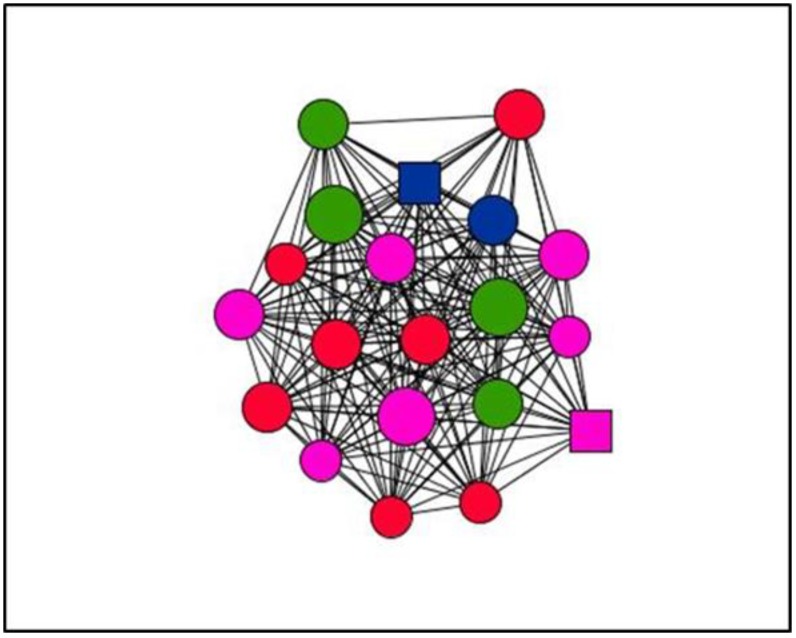
Graph Case 24. Woman, 53 years old, 3 years with chronic pain.

**Figure 3:**
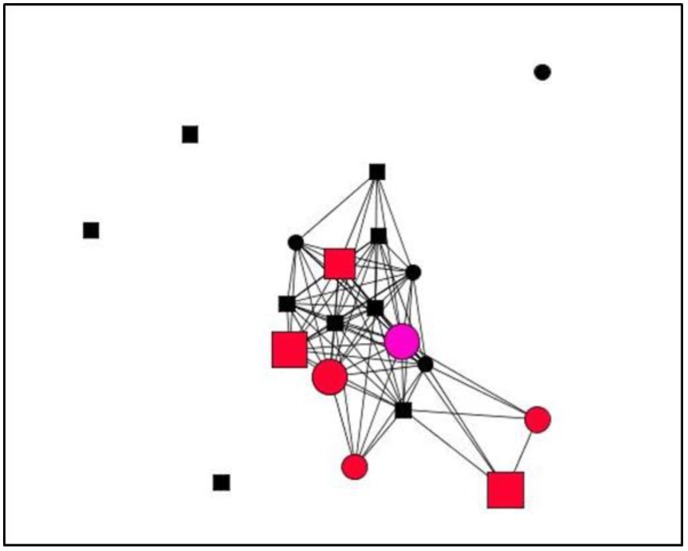
Graph Case 7. Man, 66 years old, 18 years with chronic pain.

**Figure 4:**
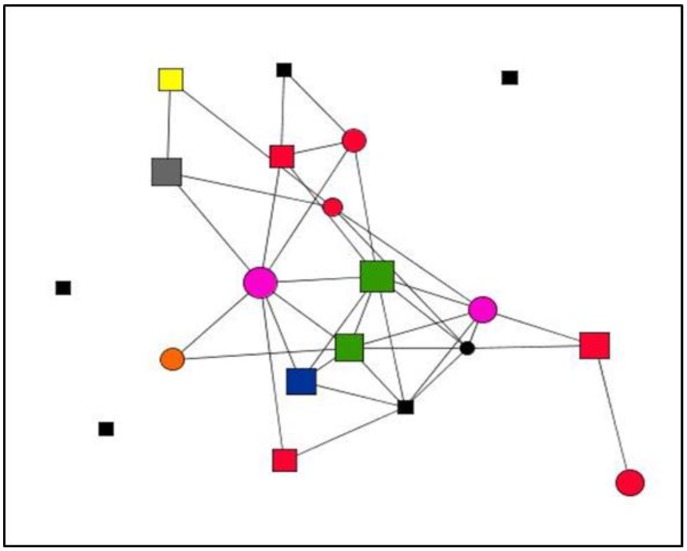
Graph Case 26. Man, 48 years old, 1 year with chronic pain.

The graphs below represent the personal support networks at ego level of two women (Figure 1 and Figure 2) and two men (Figure 3 and Figure 4), showing the ties between their contacts. In addition, the shape, colour and size of nodes indicate sex, type and satisfaction with the support received for each of the ties (see Table 4). Table 5 gives the quantitative results for social support variables in each of the egos presented. In line with the results obtained for structure according to the sex of ego for the entirety of ties (Table 1), the graphs serve to illustrate these differences at ego level. Thus, the graphs corresponding to women, indicate greater cohesion and less fragmentation than those corresponding to men as they show higher values for density and degree centrality and a lower number of components and isolates (Table 6).

The results presented here regarding the structure, composition and social support function of all the personal networks studied, together with contextual factors obtained using a qualitative approach, show the relational and social reality of people with chronic pain.

## 4. Discussion

This paper has focused on demonstrating the usefulness of Personal Network Analysis to study social support in a specific context of illness: chronic pain. The study design enabled us to obtain data on the composition and structure of personal networks from which social support emerges, for all ties studied and at ego-level. It has proved equally relevant to obtain functional social support data on providers and to capture non-providers and their characteristics.

Among the 600 relationships studied, emotional support was the most prevalent, being present in more than half of the ties analysed (63.6%), followed by this latter combined with instrumental support (21%). The most common channel of support was physical presence (47.9%), followed by this latter combined with telephone contact (33.4%) and telephone contact alone (15.7%). The most common frequency with which informants reported receiving support from their alters was weekly (36.2%) followed by daily (30.4%). As regards variations over time, the support provided by alters remained the same in the vast majority of cases (75%), while increases and decreases were observed in similar proportions (13.7% and 11.3%, respectively). Reciprocal support relations (ego has provided support to alter) were present in the majority of the relationships (76.3%). In terms of satisfaction with support received (assessed through five categories ranging from very satisfactory to very unsatisfactory), 33.2% of unsatisfactory relationships corresponded to non-providers, and 12.3% were rated as very satisfactory.

Many earlier studies on social support have paid most attention to family members as providers. Nevertheless, it is also necessary to consider other social roles such as friends and neighbours in the provision of certain types of support [43,44,45]. In this respect, the results of this study indicate the importance of friends and neighbours (215 of 600 alters) in relation to close family members (167 of 600) as potential providers of support, especially among older adults as a result of the social network changes associated with age [27,31,46]. Some authors have stressed the importance of other relational characteristics in the provision of social support, such as frequency of contact or strength of the tie [47], the influence of geographical distance [48,49,50,51], especially, concerning instrumental or tangible support since its provision requires face-to-face contact, the nature of the problem and the cultural context in which the social support operates [17,52].

Other authors have emphasised the importance of capturing qualitative aspects of social support networks [53], to help to understand the elements involved in the process. In the present research, informants’ accounts revealed some notable aspects in the provision of support by others, such as social understanding, the capacity to empathise with the situation, geographical distance and the quality of the personal relationship, aspects which affected not only the amount but also the quality of social support. Regarding support of non-providers, these results indicate that one-third of the alters did not provide support. Thus, the networks studied contained a mean of 13 providers and 7 non-providers of support. Notable among the characteristics of the latter was the predominance of men compared to women, while in terms for age, they included both children and older adults, a finding that can be attributed to the characteristics and/or difficulties of these ages as regards offering support. About the type of tie, non-providers were present in all the social roles studied except that of parents, and included close family members such as spouses, siblings and children. The qualitative interviews also yielded the same results. In this respect, the biopsychosocial model of pain [1], provides a framework for these results since it considers the impact of pain on an individual’s social dimension, including family relations, as previous studies have shown [3,4,54]. The context and reasons that explain the non-provision of support are of particular interest. Some studies have focused on the negative side of social relations, including family members and their impact on health and social support [55,56,57,58,59], highlighting the importance of the presence of non-providers in a personal network, but also of negative relations in chronic pain context.

Another suitable social support resource is the working environment, considering the impact and consequences of chronic pain in this field. At European level, a total of 19% of people with chronic pain lost their job, 16% changed job responsibilities and 13% changed jobs entirely as a consequence of their pain [3]. Given the above, it is necessary to consider the importance of relationships with co-workers as sources of social support due to their impact on job satisfaction, productivity and work adjustment and their capacity to mitigate the adverse effects of job-related stress and cushion the effect of deciding to leave work [60,61,62]. Our results indicate that almost half of the co-workers present in the networks studied were non-providers of support, a significant result for future studies focusing on support relations in this environment.

In terms of structure and composition, the metrics of centrality studied at alter level between non-providers and the total number of alters indicate that the former occupy positions with less centrality (degree and betweenness centrality) than the total. These results may reflect the informants’ long experience of chronic pain, which may have contributed to reducing social participation, weakening social ties and isolating them, as shown by the interviews, showing networks with fewer active and functionally operational contacts who offer support. 

Future research might consider the variables that influence the social support provided by social relationships considering both the size and the formal structure of the social networks in which it operates and its functional content [63] as well as the negative or conflicting aspects of relationships. Another line of interest in research on social support or informal care in the context of chronic illness and, in particular, in chronic pain might also consider the situation and experience from the standpoint of the provider of informal care. In this respect, the literature on caregivers has demonstrated the influence of experiences of care on caregivers’ affective states [64], revealing that social isolation is a risk factor for caregiver burden [65] and that social support received by caregivers is a significant predictor of the same [66]. Also, future research might study the social life of people with chronic pain, relational changes over time as a result of the experience of pain, as variations in support according to age and sex.

### Limitations

One limitation of the present study concerned the difficulty of obtaining data. The highly complex situation of general health and pain in some cases resulted in more refusals to participate in the study than expected at the outset. Regarding methodology, the number of cases and the type of sampling rendered it impossible to obtain results stratified by criteria such as age or sex, two key variables to consider in chronic pain, as shown in previous studies on prevalence.

## 5. Conclusions

The analysis of personal networks combined with qualitative techniques represents a novel and useful approach to study social support. It allows a detailed examination of related variables from a network perspective, while visualisation and network interviews yield qualitative information on the individual’s social and relational reality in the context of chronic pain. This approach does not replace but rather complements the attributional measures that have traditionally been used to assess social support and for which there is empirical evidence. Thus, it helps to elucidate factors that explain the different contexts in which support is offered and received, mainly due to the emphasis placed on the structure and composition of personal networks from which social support emerges. Finally, varied social and relational environments of people with chronic pain must be considered especially in those cases in which informal support resources are insufficient or inadequate in order to complement them with the existing formal resources at the community level.

## Figures and Tables

**Table 1:** Personal network structure variables.

		Mean	Median	SD	Min.	Max.
Density	Men	0.4	0.4	0.2	0.1	0.7
	Women	0.5	0.4	0.2	0.2	1
	**Total**	**0.6**	**0.4**	**0.2**	**0.1**	**1**
Degree centrality	Men	8.2	8.0	3.3	2.6	13.5
	Women	9.0	7.6	4.3	3.10	18.1
	**Total**	**8.6**	**8**	**3.8**	**2.6**	**18.1**
Betweenness centrality	Men	3.7	3.5	2.8	0.1	10.8
	Women	4.3	4.3	2.0	0.5	8.8
	**Total**	**4**	**3.9**	**2.4**	**0.1**	**10.8**
Components	Men	2.9	2.0	2.6	1.0	11.0
	Women	2.2	2.0	1.6	1.0	6.0
	**Total**	**2.5**	**2.0**	**2.2**	**1.0**	**11.0**
Isolates	Men	1.3	0.00	2.5	0.0	9.0
	Women	0.9	0.0	1.4	0.0	5.0
	**Total**	**1.1**	**0.0**	**2**	**0**	**9**

**Table 2:** Descriptive analysis of social support in personal networks.

Variable	Category	N (%)
**Characteristics of personal relationships in terms of social support (N = 600)**		
Satisfaction	Very satisfactory	74 (12.3)
	Quite satisfactory	132 (22)
	Satisfactory	156 (26)
	Quite unsatisfactory	39 (6.5)
	Very unsatisfactory	199 (33.2)
Variation	No change	450 (75)
	Has increased	82 (13.7)
	Has decreased	68 (11.3)
Reciprocity	Yes	458 (76.3)
	No	142 (23.7)
**Characteristics of social support in provider relationships (N = 401)**		
Type	Emotional	255 (63.6)
	Instrumental	16 (4.0)
	Informational	5 (1.2)
	All three types of support	22 (5.5)
	Emotional and instrumental	84 (21)
	Emotional and informational	14 (3.5)
	Instrumental and informational	1 (0.2)
	Health professionals	4 (1)
Frequency	Daily	122 (30.4)
	Weekly	145 (36.2)
	Fortnightly	47 (11.7)
	Monthly	57 (14.2)
	Every 2–3 months	16 (4.0)
	Every 3 months or so	14 (3.5)
Channel of transmission	In person	192 (47.9)
	Telephone	63 (15.7)
	Internet	8 (2)
	In person and telephone	134 (33.4)
	Telephone and internet	4 (1)

**Table 3:** Node degree and betweenness centrality. Total and non-provider.

	Mean	Median	SD	Min.	Max.
Non-providers: degree centrality	7.3	8	4.8	0	18
Degree centrality (total)	8.6	9	5.4	0	19
Non-providers: betweenness centrality	2.1	0.2	5.3	0	38.7
Betweenness centrality (total)	4	0.33	10.1	0	114.3

**Table 4:** Legend of graphs.

Node Shape: Sex	Node Size: Satisfaction	Node Colour: Type of Social Support	
Circle: woman Square: man	Large: more satisfaction Small: less satisfaction	Emotional: red Instrumental: blue Informational: yellow All types: green	Non-providers: black Emotional and instrumental: pink Emotional and informational: orange Instrumental and informational: grey

**Table 5:** Descriptive analysis of social support at ego level.

Variables	Categories	Case 2 N (%)	Case 24 N (%)	Case 7 N (%)	Case 26 N (%)
**Satisfaction**	Very satisfactory	3 (15)	3 (15)	4 (20)	2 (10)
	Quite satisfactory	-	10 (50)	1 (5)	6 (30)
	Satisfactory	7 (35)	7 35)	2 (10)	5 (25)
	Quite unsatisfactory	5 (25)	-	-	1 (5)
	Very unsatisfactory	5 (25)	-	13 (65)	6 (30)
**Variation**	No change	7 (35)	18 (90)	17 (85)	12(60)
	Has increased	10 (50)	1 (5)	1(5)	8 (40)
	Has decreased	3 (15)	1 (5)	2(10)	-
**Reciprocity**	Yes	18 (90)	18 (90)	10 (50)	11 (55)
	No	2 (10)	2 (10)	10 (50)	9 (45)
**Types of support**	Emotional	13 (65)	7 (35)	6 (30)	6 (30)
	Instrumental	-	2 (10)	-	1 (5)
	Informational	-	-	-	1(5)
	All three types of support	-	4 (20)	-	2 (10)
	Emotional and instrumental	2 (10)	7 (35)	1 (5)	2 (10)
	Emotional and informational	-	-	-	1(5)
	Instrumental and informational	-	-	-	1(5)
	Health professionals	-	-	-	-
	Non-Providers	5 (25)	-	13 (65)	6 (30)
**Frequency**	Daily	3 (15)	10 (50)	5 (25)	4 (20)
	Weekly	5 (25)	10 (50)	2 (10)	6 (30)
	Fortnightly	2 (10)	-	-	1 (5)
	Monthly	5 (25)	-	-	1 (5)
	Every 2–3 months	-	-	-	1 (5)
	Every 3 months or so	-	-	-	1 (5)
	Non-providers	5 (25)	-	13 (65)	6 (30)
**Channel of transmission**	In person	2 (10)	13 (65)	1 (5)	10 (50)
	Telephone	12 (60)	1 (5)	1 (5)	1 (5)
	Internet	1 (5)	-	-	-
	In person and telephone	-	6 (30)	5 (25)	1(5)
	Telephone and internet	-	-	-	2 (10)
	Non-providers	5 (25)	-	13 (65)	6 (30)

**Table 6:** Structural measures of personal networks.

	Case 2	Case 24	Case 07	Case 26
Density	0.6	1	0.4	0.2
Degree centrality (mean)	10.4	18.1	8.2	3.6
Betweenness centrality (mean)	4.3	0.5	1.9	8
Components	1	1	5	4
Isolates	0	0	4	3

## References

[1] Dueñas M., Ojeda B., Salazar A., Mico J.A., Failde I. (2016). A review of chronic pain impact on patients, their social environment and the health care system. J. Pain Res..

[2] Johannes C.B., Le T.K., Zhou X., Johnston J.A., Dworkin R.H. (2010). The Prevalence of Chronic Pain in United States Adults: Results of an Internet-Based Survey. J. Pain.

[3] Breivik H., Collett B., Ventafridda V., Cohen R., Gallacher D. (2006). Survey of chronic pain in Europe: Prevalence, impact on daily life, and treatment. Eur. J. Pain.

[4] Dueñas M., Salazar A., Ojeda B., Fernández-Palacín F., Micó J., Torres L., Failde I. (2015). A Nationwide Study of Chronic Pain Prevalence in the General Spanish Population: Identifying Clinical Subgroups through Cluster Analysis. Pain Med..

[5] International Association for the Study of Pain. http://www.iasp-pain.org/Education/Content.aspx?ItemNumber=1698#Pain.

[6] Cobb S. (1976). Social support as a moderator of life stress. Psychosom. Med..

[7] Williams P., Barclay L., Schmied V. (2004). Defining social support in context: A necessary step in improving research, intervention, and practice. Qual. Health Res..

[8] Cassel J. (1976). The contribution of the social environment to host resistance. Am. J. Epidemiol..

[9] Berkman L.F., Glass T., Brissette I., Seeman T.E. (2000). From social integration to health: Durkheim in the new millennium. Soc. Sci. Med..

[10] Lin N., Dean A., Ensel W.M. (1981). Social support scales: A methodological note. Schizophr. Bull..

[11] House J.S., Umberson D., Landis K.R. (1988). Structures and processes of social support. Annu. Rev. Sociol..

[12] Langford C., Bowsher J., Maloney J., Lillis P. (1997). Social support: A conceptual analysis. J. Adv. Nurs..

[13] Jacobson D.E. (1986). Types and timing of social support. J. Health Soc. Behav..

[14] Wills T.A., Cohen S., Syme S.L. (1985). Supportive Functions of Interpersonal Relationships. Social Support and Health.

[15] Barrera M. (1986). Distinctions between Social Support Concepets, Measures, and Models. Am. J. Community Psychol..

[16] Wills T.A., Shinar O., Cohen S., Underwood L.G., Gottlieb B.H. (2000). Measuring Perceived and Received Social Support. Social Support Measurement and Intervention: A Guide for Health and Social Scientists.

[17] Jacobson D. (1987). The Cultural Context of Social Support and Support Networks. Med. Anthropol. Q..

[18] Gottlieb B.H., Bergen A.E. (2010). Social support concepts and measures. J. Psychosom. Res..

[19] Luke D.A., Harris J.K. (2007). Network analysis in public health: History, methods, and applications. Annu. Rev. Public Health.

[20] Schoen W., Moreland-Russell S., Prewitt K., Carothers B.J. (2014). Social network analysis of public health programs to measure partnership. Soc. Sci. Med..

[21] Perkins J.M., Subramanian S.V., Christakis N.A. (2015). Social networks and health: A systematic review of sociocentric network studies in low- and middle-income countries. Soc. Sci. Med..

[22] Zhang S., de la Haye K., Ji M., An R. (2018). Applications of social network analysis to obesity: A systematic review. Obes. Rev..

[23] Schaefer D.R., Simpkins S.D. (2014). Using social network analysis to clarify the role of obesity in selection of adolescent friends. Am. J. Public Health.

[24] Valente T. (2012). Network interventions. Science.

[25] Latkin C.A., Knowlton A.R. (2015). Social Network Assessments and Interventions for Health Behavior Change: A Critical Review. Behav. Med..

[26] Pinto R.M. (2006). Using social network interventions to improve mentally ill clients’ well-being. Clin. Soc. Work J..

[27] Ashida S., Heaney C.A. (2008). Differential associations of social support and social connectedness with structural features of social networks and the health status of older adults. J. Aging Health.

[28] Hawe P., Webster C., Shiell A. (2004). A glossary of terms for navigating the field of social network analysis. J. Epidemiol. Community Health.

[29] Vassilev I., Rogers A., Kennedy A., Wensing M., Koetsenruijter J., Orlando R., Portillo M.C., Culliford D. (2016). Social network type and long-term condition management support: A cross-sectional study in six European countries. PLoS ONE.

[30] Shiovitz-Ezra S., Litwin H. (2012). Social network type and health-related behaviors: Evidence from an American national survey. Soc. Sci. Med..

[31] Harasemiw O., Newall N., Shooshtari S., Mackenzie C., Menec V. (2018). From Social Integration to Social Isolation: The Relationship Between Social Network types and perceived availability of social support in a national sample of older canadians. Res. Aging.

[32] Park S., Kang J.Y., Chadiha L.A. (2018). Social Network Types, Health, and Health-Care Use among South Korean Older Adults. Res. Aging.

[33] Christakis N.A., Fowler J.H. (2013). Social contagion theory: Examining dynamic social networks and human behavior. Stat. Med..

[34] Smith K.P., Christakis N.A. (2008). Social networks and health. Annu. Rev. Sociol..

[35] McCarty C. (2002). Structure in Personal Networks. J. Soc. Struct..

[36] Hâncean M., Molina J.L., Lubbers M.J. (2016). Recent Advancements, Developments and Applications of Personal Network Analysis. Int. Rev. Soc. Res..

[37] Tardy C. (1985). Social support Measurement. Am. J. Community Psychol..

[38] O’Reilly P. (1988). Methodological issues in social support and social network research. Soc. Sci. Med..

[39] Borgatti S.P., Everett M.G. (2013). Analyzing Social Networks.

[40] McCarty C., Molina J.L., Aguilar C., Rota L. (2007). A Comparison of Social Network Mapping and Personal Network Visualization. Field Methods.

[41] Kennedy D.P., Green H.D., McCarty C., Tucker J.S. (2011). Nonexperts’ Recognition of Structure in Personal Network Data. Field Methods.

[42] Molina J.L. (2005). El estudio de las redes personales: Contribuciones, métodos y perspectivas. Emp. Rev. Metodol. Cienc. Soc..

[43] Miller R.J., Darlington Y. (2002). Who supports? The providers of social support to dual-parent families caring for young children. J. Community Psychol..

[44] Li H., Ji Y., Chen T. (2014). The roles of different sources of social support on emotional well-being among Chinese elderly. PLoS ONE.

[45] Hough E.S., Magnan M.A., Templin T., Gadelrab H.F. (2005). Social network structure and social support in HIV-positive inner city mothers. J. Assoc. Nurses AIDS Care.

[46] Feld S.L., Suitor J.J., Gartner J. (2007). Describing Changes in Personal Networks over Time. Field Methods.

[47] Herz A. (2015). Relational constitution of social support in migrants’ transnational personal communities. Soc. Netw..

[48] Fernández M. (2012). Social support networks in Spain: The factors that determine models of choice. Int. Sociol..

[49] Mok D., Wellman B. (2007). Did distance matter before the Internet? Interpersonal contact and support in the 1970s. Soc. Netw..

[50] Weiner A.S.B., Hannum J.W. (2012). Differences in the quantity of social support between geographically close and long-distance friendships. J. Soc. Pers. Relat..

[51] Seeman T.E., Berkman L.F. (1988). Structural characteristics of social networks and their relationship with social support in the elderly: Who provides support. Soc. Sci. Med..

[52] Pearlin L.I., Cohen S.L., Syme S.L. (1985). Social structure and processes of social support. Social Support and Health.

[53] Agneessens F., Waege H., Lievens J. (2006). Diversity in social support by role relations: A typology. Soc. Netw..

[54] Strunin L., Boden L.I. (2004). Family consequences of chronic back pain. Soc. Sci. Med..

[55] DeLongis A., Capreol M., Holtzman S., O’Brien T., Campbell J. (2004). Social Support and Social Strain Among Husbands and Wives: A Multilevel Analysis. J. Fam. Psychol..

[56] Rook K.S. (1984). The negative side of social interaction: Impact on psychological well-being. J. Pers. Soc. Psychol..

[57] Rook K.S. (2003). Exposure and reactivity to negative social exchanges: A preliminary investigation using daily diary data. J. Gerontol..

[58] Sapin M., Widmer E., Iglesias K. (2016). From support to overload: Patterns of positive and negative family relationships of adults with mental illness over time. Soc. Netw..

[59] Newsom J.T., Rook K.S., Nishishiba M., Sorkin D.H., Mahan T.L. (2005). Understanding the relative importance of positive and negative social exchanges: Examining specific domains and appraisals. J. Gerontol..

[60] Baruch-Feldman C., Brondolo E., Ben-Dayan D., Schwartz J. (2002). Sources of social support and burnout, job satisfaction, and productivity. J. Occup. Health Psychol..

[61] Kim H., Stoner M. (2008). Burnout and turnover intention among social workers: Effects of role stress, job autonomy and social support. Adm. Soc. Work.

[62] Nissly J., Barak M.M., Levin A. (2004). Stress, Social Support, and Workers’ Intentions to Leave Their Jobs in Public Child Welfare. Adm. Soc. Work.

[63] House J.S. (1987). Social Support and Social Structure. Sociol. Forum.

[64] Robertson S.M., Zarit S.H., Duncan L.G., Rovine M.J., Femia E.E. (2007). Family caregivers’ patterns of positive and negative affect. Fam. Relat..

[65] Adelman R.D., Tmanova L., Delgado D., Dion S., Lachs M.S. (2014). Caregiver Burden: A Clinical Review. J. Am. Med. Assoc..

[66] Rodakowski J., Skidmore E.R., Rogers J.C., Schulz R. (2012). Role of social support in predicting caregiver burden. Arch. Phys. Med. Rehabil..

